# Macroscopic Skin Examination Can Determine the Number of Strips Necessary to Study the *stratum corneum* in Dogs

**DOI:** 10.3390/vetsci10090547

**Published:** 2023-09-01

**Authors:** Marion Mosca, Mélanie Legain, Guillaume Noël, Adrien Idée, Didier Pin

**Affiliations:** 1 Dermatology Department, Interaction Cell Environment, VetAgro Sup, Université de Lyon, UPSP 2016.A104, 69280 Marcy l’Etoile, France; melanie.legain@vetagro-sup.fr (M.L.); adrien.idee@vetagro-sup.fr (A.I.); 2 Institut Claude Bourgelat ICLB, VetAgro Sup, Université de Lyon, 69280 Marcy l’Etoile, France; guillaume.noel@vetagro-sup.fr

**Keywords:** skin barrier, non-invasive measurement method, stratum corneum, atopic dermatitis

## Abstract

**Simple Summary:**

The skin barrier is the cornerstone of skin function. Evaluations of its morphological aspects, protein content and gene expression are necessary to study atopic dermatitis. Many human studies have assessed the presence of biomarkers in the skin of atopic patients. In dogs, previous biomarker studies have been performed using skin biopsies, which are invasive. This prospective blinded experimental pilot study aimed to evaluate the relevance of a noninvasive technique to extract information from the *stratum corneum* in dogs with the goal of facilitating future studies on atopic dermatitis.

**Abstract:**

To evaluate the skin barrier, the *stratum corneum* (SC) must be isolated and extracted. Currently, skin biopsy is the gold standard method to investigate skin immunology and the presence of biomarkers in dogs. However, a standardized, non-invasive tool to exclusively remove the SC would be of great interest to study healthy and atopic dogs. In this study, we performed D-squames^®^ tape stripping with standardized pressure on seven healthy beagle dogs. A control site was defined and then 25 strips, 50 strips and as many strips as needed to achieve a shiny appearance of the skin were performed on three different experimental sites. After stripping, blinded histopathological examination of a skin biopsy from each site was performed. The number of tape strips required for the skin to become shiny varied between individuals, with a mean of 40 (29–50) strips. There was no significant difference in SC depth between the control site and the site that underwent 25 tape strips. In contrast, the use of 50 strips removed almost all of the SC, with a mean remaining SC depth of 7.82 µm. These data suggest that this non-invasive method can effectively remove the SC, with individual variability, and that a shiny appearance of the skin after stripping can be used as an accurate marker of SC removal.

## 1. Introduction

The skin barrier is an essential component of normal skin function and is dysregulated in a number of skin diseases in humans and animals. Therefore, it is very important to characterize the skin barrier to be able to describe and better understand the pathogenesis of skin diseases. Atopic dermatitis is a very common skin condition characterized by skin barrier dysfunction, with an estimated prevalence of up to 10% in dogs [1]. Moreover, atopic dogs are a relevant model for human atopic dermatitis because the epidemiology and clinical lesions in spontaneously affected dogs are very similar to those in humans [2]. In both species, the immunopathogenesis of atopic dermatitis involves a mixed inflammatory response, with Th1, Th2, Th17 and Th22 responses [3,4]. Microbiome studies also show many similarities between dogs and humans affected by atopic dermatitis [5]. Human studies have evaluated biomarkers to better understand the pathogenesis of atopic dermatitis, predict disease development and target certain cytokines to develop novel targeted therapeutics [6]. Several studies on models of atopic dermatitis in dogs have also investigated different immune mediators present in skin biopsies [7].

In both humans and dogs, skin biopsy is the gold standard sampling technique to study skin immunology, but an increasing number of studies are based on the use of noninvasive techniques to repeat sampling without pain or scarring [8]. Tape stripping is a reliable, noninvasive tool to study inflammatory biomarkers in the *stratum corneum* (SC) [9,10,11,12,13]. Tape strips have been used to study the morphological appearance and number of corneocytes in humans [14,15], healthy dogs and cats, and dogs suffering from atopic dermatitis [16]. This noninvasive method has also been used for penetration studies of toxicologically or pharmaceutically relevant substances through the SC [17,18,19], assessments of the cosmetic effect of different products [20], analyses of epidermal biomarkers in inflammatory skin diseases in humans [6,8,9,10,12,21] and studies of the microbiome [22]. Furthermore, tape stripping has been used to evaluate barrier defects in atopic dermatitis in humans [9,12,23].

The objectives of this prospective blinded pilot study were to evaluate and standardize a noninvasive tape stripping technique in dogs to facilitate the exploration of biomarkers in canine atopic dermatitis. The specific aim was to determine the number of strips needed to remove the SC in dogs.

## 2. Materials and Methods

This study was conducted in compliance with EU Directive 2010/63/EU for animal experiments and with ARRIVE guidelines. The study protocol was approved by the VetAgro Sup Ethics Committee (French ethical committee number 18) and authorized by the French Ministry of Higher Education and Research under Project Number APAFIS: 2245V2.

### 2.1. Study Population

Seven healthy male beagle dogs aged between 28 and 30 months (mean = 29.4 months) and with a mean weight of 9.2 kg were included in this study. These dogs were living outdoors at the Claude Bourgelat Institute. Experiments were performed inside under controlled environmental parameters.

### 2.2. Stratum Corneum Sampling

**Figure 1:**
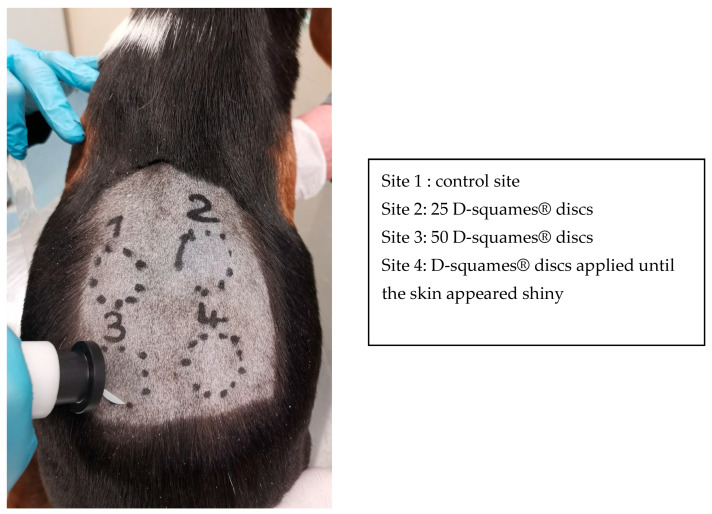
Back of a healthy beagle dog with four different experimental sites. A D-squame^®^ disc is present on Site 3 and the D-squame Pressure Instrument is ready to use.

Four different 3 cm diameter circular areas were outlined on the back of each dog and the SC was collected by tape stripping (Figure 1). Circular adhesive tapes (22 mm diameter, 3.8 cm^2^, D-squames^®^ discs; Monaderm, Monaco, France) were placed and pressed for 5 s with a pressure of 225 g.cm^2^ using a D-squame Pressure Instrument D500 (Monaderm, Monaco, France). The tape was then removed using tweezers in a quick uniform movement, following the longitudinal axis of the back. Between 0 and 25 consecutive tapes were applied on the same skin area, and a new tape was used for each application. Site 1 was a control site without stripping, and 25 and 50 D-squames^®^ discs were applied to Site 2 and Site 3, respectively. At Site 4, D-squames^®^ tapes were applied until the skin appeared macroscopically shiny but before oozing started. A slight erythema was sometimes associated with the region of tape stripping.

### 2.3. Evaluation of the Depth of Remaining Stratum Corneum

**Figure 2:**
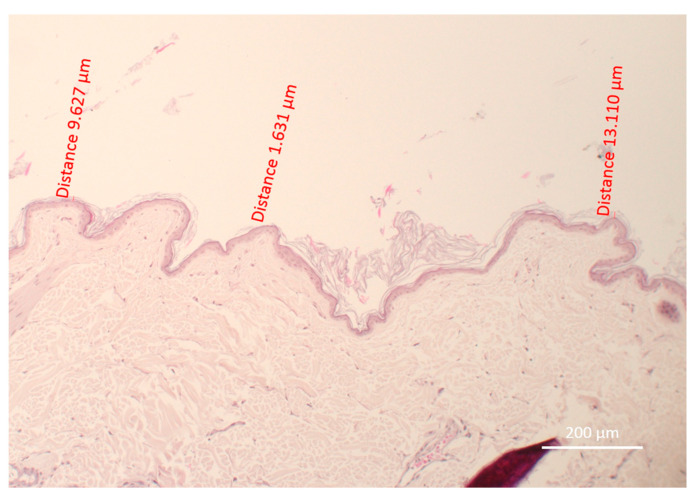
Measurements of the thickness of remaining stratum corneum at Site 4 of Dog 4. The three zones with the thinnest stratum corneum were chosen and measured using Zen^®^ software (Version 2.0.0.11). The mean thickness was 8.12 µm.

Skin biopsies were performed at the center of each site via 6 mm punch biopsy. The biopsy specimens were fixed, stained with hematoxylin and eosin, then observed microscopically and analyzed in ZEN^®^ software by a blinded operator. Four sites were compared for each dog. Within the regions with the thinnest skin on each biopsy, three different measurements were made by drawing a line using the ZEN^®^ software to determine the thickness of the remaining keratin layers in micrometers. All measurements were performed at the same magnification (×10, Figure 2).

### 2.4. Statistical Analysis

Statistical analysis was performed using GraphPad Prism 9.0.0, and *p*-values less than or equal to 0.05 were considered significant. Shapiro–Wilk tests were used for normality testing, and the Friedman test was performed to evaluate the difference in means among the sites.

## 3. Results

### 3.1. An Increased Number of D-squames^®^ Discs Is Associated with a Decreased Thickness of Remaining Stratum Corneum Layers

**Figure 3:**
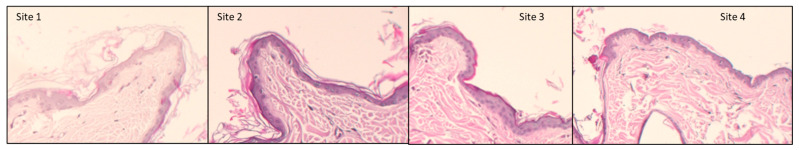
Differences among the four sites on Dog 3, H&E, ×200.

Microscopic evaluation of the thickness of the SC was performed by a histopathological examination of skin biopsies. The thickness of the SC layers decreased as the number of tape strips increased at the experimental sites (Table 1): the SC was thickest to thinnest for Site 1 (0 tape strips) > Site 2 (25 tape strips) > Site 4 (mean 40 tape strips) > Site 3 (50 tape strips). Site 1, the control site, had the thickest SC. Site 3, which underwent the most tape strips (50), had the least visible SC layers (Figure 3) and was characterized by only a *stratum compactum* layer, except in the skin folds.

### 3.2. There Was No Significant Difference between Sites 1 and 2 or between Sites 3 and 4

**Figure 4:**
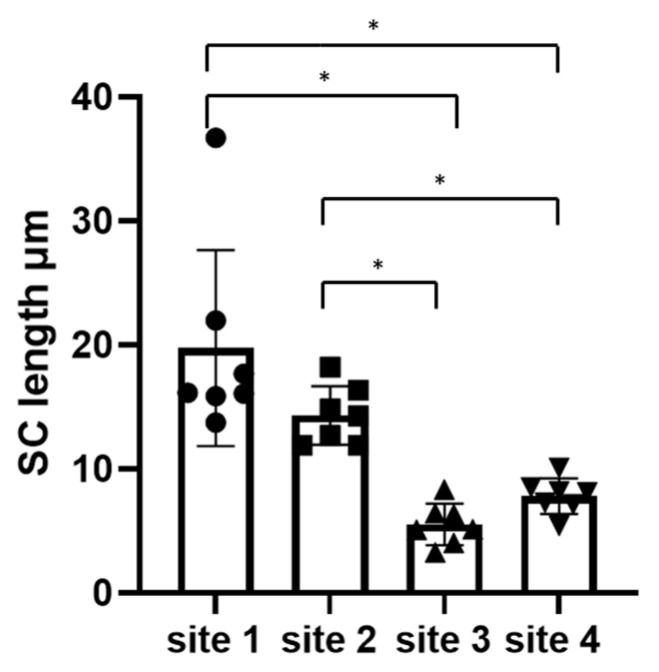
Mean thickness of the remaining *stratum corneum* layers in micrometers at each site on the seven dogs. Friedman test, * indicates *p* < 0.05.

The mean values of the remaining SC thickness are presented in Table 1. The SC thickness was not significantly different between Site 1 and Site 2, which suggests that using 25 D-squames^®^ discs is not sufficient to remove all of the SC. Similarly, the SC thickness was not significantly different between Site 3 and Site 4. This suggests that using sufficiently many tape strips to make the skin shiny can remove a similar quantity of SC to using 50 consecutive strips (Figure 4). Significant differences in SC thickness were also found between Site 1 and Site 3, between Site 1 and Site 4, between Site 2 and Site 3, and between Site 2 and Site 4 (Figure 4).

The mean of three measurements is shown for each site on each dog.

## 4. Discussion

In our prospective blinded experimental study, we showed that consecutive stripping with D-squames^®^ discs until the skin becomes macroscopically shiny removes most of the SC, leaving a mean thickness of 7.82 µm. This thickness was not statistically different from that obtained with a fixed number of 50 consecutive D-squames^®^ discs, which left only the *stratum compactum* on histopathological examination. However, the number of D-squames^®^ discs required to create a shiny appearance of the skin varied from 29 to 50 among the individuals. This may reflect the interindividual variability in SC thickness.

Previous studies have shown that acute barrier disruption by adhesive tapes is influenced by pressure, time and anatomical location [24]. Therefore, the aim of our study was to evaluate the feasibility of a standardized D-squames^®^ technique. Our results suggest that it is not possible to define a specific number of D-squames^®^ discs required to remove the SC in all dogs, but rather that a macroscopic skin evaluation of the skin allows researchers to assess the depth of sampling. Our data suggest that a shiny appearance of the skin is a reliable marker of SC removal by tape stripping and that the number of tape strips required for the skin to become shiny will likely vary among individuals.

The D-squames^®^ technique has numerous advantages when compared with skin biopsies, including no pain, repeatability on the same site for follow-up, no scarring and restriction to the epidermis. This is in contrast to skin biopsies, which contain a mix of epidermal and dermal structures and may even include subcutaneous tissue. Furthermore, one study showed that results using the D-squames^®^ technique correlated better with severity of skin lesions in atopic dermatitis than the results of skin biopsies [10].

Although the D-squames^®^ method has not been previously used in dogs to remove the entire SC, it has been characterized in humans and pigs. The number of tape strips needed for SC removal varies widely in human studies. This is because the SC thickness varies between individuals [25] and the weight of SC removed decreases exponentially with each consecutive tape strip [26]. Moreover, there are differences in the number of strips required to remove the entire SC when using various types of tape. A previous study showed that at least 30 consecutive D-squames^®^ discs were required to remove the SC, whereas only 15 consecutive tape strips were sufficient when Barrier tape was used [27]. Another study showed that on the flexor surface of the forearm, approximately 30 tape strips were needed to strip off most of the SC [14]. A separate study, using reflectance confocal microscopy and Squame scan, found that the SC was completely removed in all human volunteers after 35 tape strips on the middle volar forearm. Advancement into the epidermis was predominantly achieved by the first 15 tape strips, which removed 25% of the total epidermis, whereas 35 tape strips removed 33% of the epidermis. In contrast, in pigs, the SC was removed linearly, at least up to 20 tape strips [28]. Each tape strip removed approximately 0.4 μm of SC, which corresponds to approximately one cell layer. The linearity was characterized by an equation that estimated that 28 D-squames^®^ discs were required to remove the SC completely. Other studies have used 6 [9], 16 [12] or 23 [21] D-squames^®^ discs to study the protein content in the skin barrier. The protein removal per tape decreased with increasing depth [29].

Together, these studies show that the number of D-squames^®^ discs needed to remove the SC is similar in humans and pigs (between 28 and 35) and lower than the 40 to 50 tape strips we needed to remove the SC in dogs. A higher number of tape strips may be required in dogs due to the high density of hair follicles in this species, which may retain more skin during each tape strip. Our results were significantly higher than those of a previous study characterizing canine barrier restoration, which showed removal of the SC histologically after 30 successive strips with Scotch^®^ Transparent Tape [30]. However, as discussed above, a similar difference was found in human studies showing that 30 consecutive D-squames^®^ discs were required to remove the same amount of SC as 15 consecutive strips with Barrier tape [27]. Furthermore, we could hypothesize that periocular, inguinal or axillary regions where the skin is thin would necessitate fewer D-squames^®^ tapes to remove the entire SC. Our finding that macroscopically shiny skin reflects the sampling depth will allow researchers to adjust the number of D-squames^®^ discs used to sample different regions. This will be of interest when studying spontaneously atopic dogs, since lesions are present at different sites.

While the D-squames^®^ technique is a promising non-invasive method to sample the SC, further studies are needed to compare the immune response markers in the SC to that in the total epidermis. Proteins and cells extracted via the D-squames^®^ technique may represent only a small part of the inflammatory response and may not be representative of the global response in atopic dermatitis and other skin diseases. Therefore, future studies should be performed to compare the results of cytokine extractions or transcriptomic analysis from samples collected by the use of D-squames^®^ discs and by skin biopsies.

## 5. Conclusions

There is a strong interest in finding methods to access internal biochemical, molecular and genetic processes through noninvasive and minimally invasive methods. Tape stripping is noninvasive, painless and nonscarring, and it allows repeated sampling. Therefore, this method may be beneficial for longitudinal canine studies and clinical trials, in which serial measures are needed to identify predictors of treatment response, disease course and comorbidities. Furthermore, tape stripping could be a valuable way to assess skin barrier biomarkers in canine atopic dermatitis. This study showed that it is possible to use the D-squames^®^ technique to extract the SC in dogs. Moreover, we found that the best way to collect and study the entire SC is to use a number of D-squames^®^ discs determined by a macroscopic evaluation of skin appearance.

## Figures and Tables

**Table 1:** Mean thickness of the remaining *stratum corneum* layers for each dog.

Thickness (µm)	Dog 1	Dog 2	Dog 3	Dog 4	Dog 5	Dog 6	Dog 7	Mean
Site 1	15.89	21.98	36.72	16.08	13.74	17.68	16.15	19.7485714
Site 2	11.91	18.22	16.36	14.85	14.28	11.93	12.72	14.3242857
Site 3	6.38	3.29	5.1	4.06	6.44	8.34	5.15	5.53714286
Site 4 (number of D-squames^®^)	8.19 (29)	5.4 (50)	7.28 (42)	8.12 (46)	8.53 (42)	10.09 (41)	7.16 (29)	7.82428571 (39.85714)

## Data Availability

Raw data are available upon reasonable request to the corresponding author.
